# Pup mortality in laboratory mice – infanticide or not?

**DOI:** 10.1186/1751-0147-55-83

**Published:** 2013-11-20

**Authors:** Elin M Weber, Bo Algers, Jan Hultgren, I Anna S Olsson

**Affiliations:** 1 Department of Animal Environment and Health, Swedish University of Agricultural Sciences,PO Box 234,532 23 Skara, Sweden; 2 Laboratory Animal Science, IBMC-Instituto de Biologia Molecular e Celular, Universidade do Porto, Rua Campo Alegre 823, 4150-180 Porto, Portugal

**Keywords:** Perinatal mortality, Maternal behaviour, Laboratory mice, Infanticide, Cannibalism

## Abstract

**Background:**

Despite being the most commonly used mammal in biomedical research, problems with perinatal mortality in mice have received little attention and the causes of pup death are still poorly known. Females are often housed alone with their litters and since the lost pups are generally eaten, it is commonly assumed that the mother has killed them. However, more detailed observations than have been reported previously in the literature are required to establish if the cause of death is infanticide. Litter loss can only be prevented efficiently after underlying causes have been carefully investigated and interpreted. The aim of this study was to investigate if females actively kill their pups by observing the behaviour of females and pups in litters that later were lost. We used video recordings of females that lost their entire litter to observe females in detail from parturition until the pups died. In total, 10 C57BL/6 females (wildtype and the knockouts Hfe^−/−^ and β2m^−/−^) were studied, housed in Makrolon II cages with or without access to a small amount of nesting material.

**Results:**

Three of the females had pups that were never seen moving, and another three females had one or two pups that never moved, indicating that some pups were most likely still-born. In five females with live-born pups, detailed observations from the time when a pup was last seen moving until it died were possible to carry out. We observed females eating dead offspring and interacting with both moving and dead pups. However, we never observed a pup stop moving when manipulated by the female, nor were any wounds seen in the pups. Hence, we found no evidence of infanticide when studying females that had lost their entire litter.

**Conclusion:**

These results suggest that other causes than infanticide plays a major role in mouse pup death, and stress the need for more systematic and careful investigations of the causality of litter loss.

## Introduction

The laboratory mouse is the most commonly used mammal in biomedical research. Successful mouse breeding is a crucial part of providing animals for research. High perinatal mortality is a relatively common problem when breeding, especially in genetically modified mice [1,2]. In a previous study we found a total mortality rate (calculated as percentage of entire litters being lost before weaning at around 21 days) of 32% for C57BL⁄6 and 20% for BALB⁄c, two of the most common strains of laboratory mice [3]. However, reported mortality rates vary greatly: from nearly 0 to 50% in experimental studies of C57BL/6 mice [4-7] compared to almost 13% reported for the same strain by a commercial breeder [8]. Litter loss leads to an increase in the number of breeding animals needed to supply experimental animals, which in turn increases costs and counteracts the 3R goal of reducing the number of animals used for experimental purposes [9]. Whether pain and suffering is involved is likely to depend on the age of the pups [10] as well as the cause of death, but large numbers of animals dying from unidentified causes is arguably a welfare problem.

Despite a high mortality, little is known about the way pups die. The practical observation of litter loss is typically that the pups are not found when the cage is first inspected after parturition, that they disappear from one day to the other, or that they are found dead or partly eaten. Females are often housed alone with their litters and since dead pups are generally eaten, it is commonly assumed that the mother has killed them. Several authors refer to infanticide [11,12] or cannibalism [13-16] as the cause of death. However, such a conclusion requires more detailed observations than can be found in literature. Infanticide refers to the killing of young by conspecifics [17,18] and cannibalism is defined as the eating of (flesh of) conspecifics [19], including both killing followed by eating and eating conspecifics already dead [18].

In a number of experimental studies, the effect of environmental factors on reproductive performance has been investigated [*e.g.*[4,20-25]. However, the method used to measure pup mortality is to compare the number of weaned pups with the number of pups born. This gives information of the survival rate, but no information on *how* the pups die.

Determining how pups die is crucial for the understanding of mortality. Only with this knowledge can pup death be efficiently prevented. Apart from being killed by the mother, factors like starvation and hypothermia can play an important role for survival in the altricial mouse. However, females often give birth during the night and parturition is seldom monitored in laboratory practice. Periparturient females are usually left undisturbed, which makes it impossible to determine the status of neonatal pups, weighing 1–2 g and being hidden in the nest. Furthermore, post mortem examination, as used by Hauschka [26], cannot be done when the pups have been eaten. Detailed descriptive studies of maternal behaviour are therefore necessary for the understanding of the potential role of the mother as well as the sequence of events leading up to pup death. To our knowledge, detailed behavioural studies of this phenomenon have not been published before. In the present study we aimed to investigate if in cases of pup mortality any pups are actively killed by their mothers.

## Material and methods

To describe maternal behaviour of relevance for infanticide, two sets of video recordings from earlier research were used (studies on maternal behaviour in female mice conducted at the Institute for Molecular and Cell Biology, Porto, Portugal). The first set was recorded in 2005 (study A) and the second one in 2006–2007 (study B). We selected females whose entire litters were lost before weaning, and carried out detailed behavioural observations of mothers and pups from birth to litter loss. Whereas it is ethically challenging to perform experimental studies of infanticide [27], and practically challenging to monitor an unpredictable phenomenon which cannot be captured reactively (by the time that litter loss is evident, it is too late to record the behaviour leading up to it), in this study we take advantage of the fact that mortality occurred unintentionally in these two studies which involved video recordings covering the relevant time period.

### Animals and housing

The animals studied were primiparous female mice of the C57BL/6 strain (study A; n = 5) or with a C57BL/6 background, knockouts Hfe^−/−^ or β2m^−/−^ (study B; n = 5). The total number of females housed in the same way in the original study was 10 (study A) and 20 (study B). Pairs of females from the same litter had been mated with one sibling male and were housed singly from approximately 14 days after mating. The females gave birth between June and September, 2005 (study A) or between August 2006 and March, 2007 (study B).

The females were housed in standard polycarbonate cages (Makrolon II, Tecniplast Italy; L×W×H 265×205×140 mm) provided with corncob bedding (study A) or corncob and half a nestlet (Lillico Biotechnology, UK; Study B) per cage. Room temperature was maintained at 19–23°C and relative humidity at 65–72%. A 12-h light: 12-h dark cycle was used with lights on at 05:00, and the animals were given standard food (Mucedola RF25, Italy) *ad libitum* and autoclaved tap water. The cages were cleaned once a week, except for the time around parturition when the females were left undisturbed until day 10 (study A) or day 4 (study B) postpartum.

The studies were carried out under a project license (ref. 003758) issued by Direcção Geral de Veterinária, the competent authority for animal protection in Portugal.

### Data collection

#### Video recordings

The animals were video recorded in their home cages from approximately 3 d before until 4 d after parturition. Four cages were recorded simultaneously using cameras (Ikegami ICD-47E, B/W CCD, Japan) connected to a time lapse recorder (Panasonic AG-TL750E, Thailand). The recordings were rotated by means of a camera switcher (Sanyo VQC 809-P, Japan) at 30 s intervals, and each cage was thus in view for total 15 min during each hour. Infrared lights (Monacor, P 1204ST, Sweden) were used during dark hours.

#### Behavioural observations

Day of birth was noted by daily visual inspections of the animals. To determine the exact time when parturition began, video recordings were scanned. After detection of pups the film was rewound and played at fast speed forward to find the female in birth position. Time for parturition was defined as the time when the first pup was delivered, or (if the pup was not seen) the first time when the female was seen in birth position [28].

The Observer XT 6.1 software (Noldus Information Technology, The Netherlands) was used for scoring behaviours, starting at the time of birth. Both scan sampling and continuous observations of certain time periods were used. In both cases, behaviours were recorded as occurring or not. Only behaviours related to interactions between mother and pups were included (Table 1). On the videos used, the pups were not marked individually and no physical examination of the pups had been carried out, in order to avoid any possible impact of handling on the pups or on mother-young interactions.

**Table 1:** Ethogram describing the behaviours observed

**Behaviour**	**Description**
*Female behaviour*	
Paw manipulation in nest	Female manipulates pup with only paws, inside nest
Paw manipulation outside nest	Female manipulates pup with only paws, outside nest
Mouth manipulation in nest	Female manipulates pup with mouth, inside nest
Mouth manipulation outside nest	Female manipulates pup with mouth, outside nest
Eating pup in nest	Female eating pup, inside nest
Eating pup outside nest	Female eating pup, outside nest
Eating	Female eating something, not possible to distinguish what
Activity in nest	Female active in nest, type of activity not possible to determine
Activity outside nest	Female active with pup outside nest, type of activity not possible to determine
Parturition	Female giving birth to pups
*Pup behaviour*	
Movement outside nest	Movement of pup outside nest
Movement inside nest	Movement of pup in nest
Still outside nest	Pup lying still outside nest
Still in nest	Pup lying still in nest

**Figure 1:**
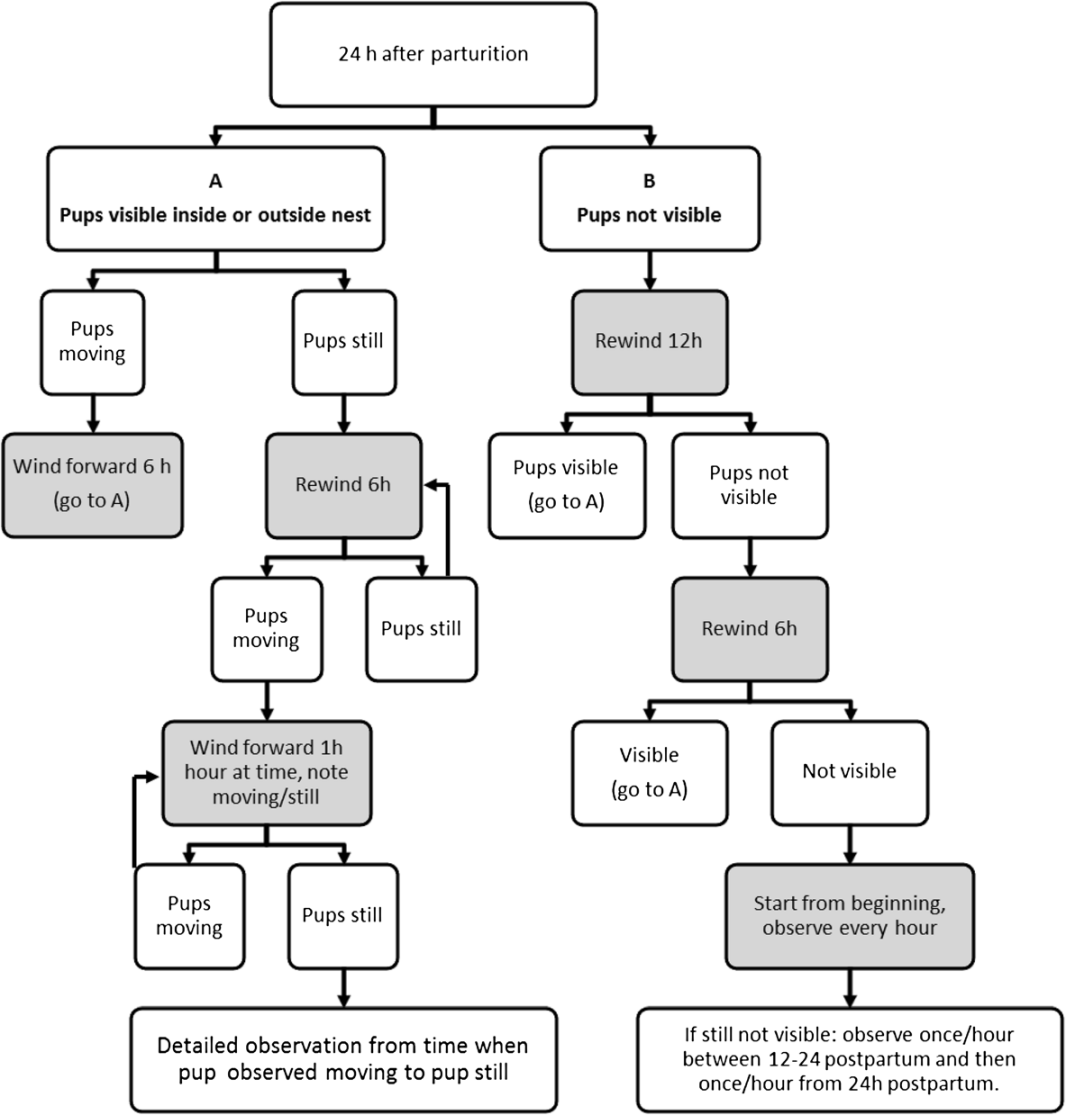
**Flowchart describing the different steps of the scanning procedure used to detect when each individual pup died.** Scanning began 24 h after parturition, and then the film was wound back *or* forward (indicated with grey boxes) depending on if the pups were moving, still or not visible.

To establish time of death, the terms “pup moving” and “pup still” were used as indicators. A pup was defined as dead when it was lying still and never seen moving again. We observed if the pups moved or not immediately after birth and 1 h postpartum. Then the female and pups were observed at certain time points, using a predefined flowchart (Figure 1), to detect when each individual pup died. During this scan, only the behaviours “pup still” and “pup movement” were recorded. Since pup movements were often difficult to detect, the animals were observed for a period of 10 min at every time point in the flowchart. If the pups were still not seen after 10 min (because they were e.g. hidden by the female in the nest, covered by nest material or sawdust) the film was observed for a longer period (1 h or more) in order to see the pups. When the behaviour ‘pup still’ was observed, the still pup was tracked backwards to observe what took place before it stopped moving. The sequence (1 h or more) from when the pup was last seen moving until it was still was observed in detail (Moving-to-Still observation, M→S), recording all behaviours in the ethogram. After M→S observation of one pup, the scanning continued to detect when the next pup stopped moving, and so on.

Of the ten females, recordings were partly missing for two females (A1: 19.5-27 h after birth; B4 36–60 h after birth).

## Results

### Time of birth

Four females gave birth during the dark period and the remaining six females between 05:30 and 11:00. We did not measure the duration of parturition, but as revealed by detailed observations, one female gave birth to a new pup 8 h after the first pup in the litter.

### Behaviour

Three of the ten females observed had pups that were never seen moving. Two of the remaining seven females with moving pups had one pup that was never seen moving, and another of these females had two pups that were never seen moving. In five of the seven females with live pups, detailed M→S observations were possible to carry out for at least one pup per female. During these observations, the behaviour “mouth manipulation with moving pup” was never followed directly by “pup still”.

**Figure 2:**
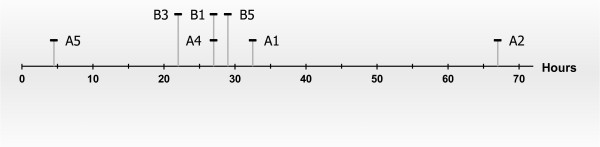
Timeline illustrating the time (in hours) from birth until pups were seen moving for the last time in four females from study A and three females from study B.

**Figure 3:**
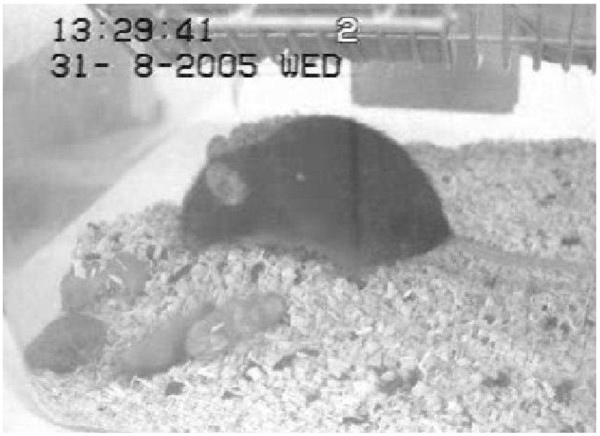
Female with four dead pups; no visual signs of wounds in the pups.

In the remaining two females, nest material, poor video quality or missing recordings made it impossible to see when the pups stopped moving. In the females with at least one live pup, time until the last pup was seen moving differed from 4.5 to 67 h after birth (Figure 2). No wounds were visually detected on intact pups during the video observations (example of dead pups in Figure 3), whereas partly eaten pups could be observed in six females.

### Detailed M→S observations

Below follows a description of the five females where detailed M→S observations were obtained (A1 refers to a female in study A, etc.). During the M→S observations, all behaviours occurring during the observation period were recorded, but the only behaviours seen in the female where “activity in nest” and “mouth manipulation in nest”. On all occasions where the female behaviour “mouth manipulation in nest” was recorded, the pups were seen moving after these interactions.

**A1:** Parturition started at 6:30, June 5; three pups were born. At 19 h after birth, three pups were seen moving. It was not possible to determine when the two first pups died (poor video quality and partly missing recordings). The third pup died sometime between 31.5 (last seen moving) and 33.5 hours (first seen still, not moving again) after birth. Just before scoring this pups’ last movement the female was manipulating the pup shortly with her mouth but the pup continued to move after the manipulation. Hereafter the female was lying still in the nest, not interacting with the pup.

**A2:** Parturition started at 22:40, August 3; three pups were born. At 41 h 40 min postpartum, all pups were seen moving, but the first pup was seen dead 20 min later. Between this pups’ last movements and the first observation of the pup still, the female was seen manipulating pups with mouth at three occasions, but the manipulated pups were seen moving after these interactions. The behaviour “activity in nest” was seen on six occasions; it was however difficult to distinguish the pups during these recordings. The second pup was seen dead at 45.5 h after birth, and only one short “activity in nest” was registered between first and second pup was observed dead. The third pup was seen moving on several occasions and was rather active. However, the amount of movement dropped over time and the last movements detected were very small. The last movement was seen at 66 h 50 min after birth; 15 min later the pup was still and partly hidden under sawdust, no interaction between mother and pup was seen during this period.

**A4:** Parturition started at 05:33, September 7; five pups were born. Two pups were never seen moving. The first time a live born pup was seen dead was 16 h after birth. The last movement detected in this pup during scanning was at 15 h 10 min after birth and after this two mouth manipulations were recorded, but the pup moved again after this manipulation before it died. The second live pup moved at 23 h 45 min after birth, and 24 min later it was dead. No interaction between female and pup was seen during this period. The last pup movement was seen at 27 h 15 min after birth and this pup was dead 2 h 40 min later. The female was mainly lying still in the nest during this period. The behaviour “activity in nest” was recorded twice for the female after the last pup movement but there was no observed mouth manipulation.

**A5:** Parturition started at 9:00, August 31; four pups were born. The pups were only seen moving on three occasions. The first pup movement was detected 1 h after birth when one pup was seen opening its mouth. After this movement the female retrieved the pup to the nest and two occasions of mouth manipulation were recorded. The pup was still during these interactions. Two hours after parturition when the female was outside the nest, another small movement was seen in one pup. The female immediately returned to the nest and a short mouth manipulation with the pup was recorded. The female was also seen active in the nest on two occasions, but then remained still in the nest until leaving it again 20 min later; there were then no pup movements. The last pup movement was seen at 4.5 h after parturition, this movement was also very small and only the pup’s head was visible. Five minutes after this movement this pup was seen still and no interaction between the female and this pup was seen during this 5-min period. It was not possible to determine if the movements described came from the same pup or three different pups.

**B1:** Parturition started 07:40, August 28; three pups were born. At 23 h after birth, two pups were seen moving inside the nest; 1 h 15 min later the female pushed one still pup to the outer edge of the nest and started to eat the pup or the placenta (not possible to distinguish) a few minutes later. It was not possible to determine if this pup had previously moved. Ten minutes later one pup moved inside the nest and another still pup was seen next to the first still pup, but this pup was seen to move again after 20 min. The last pup movement was seen at 26 h 45 min after birth. Hereafter the female spent 4 h lying still in the nest, leaving it only very shortly. The pups were difficult to see and it was not possible to determine the exact time when each pup stopped moving, but at 31 h 10 min after birth two dead pups and one partly eaten pup was seen in the outer edge of the nest.

### Additional observations

**Figure 4:**
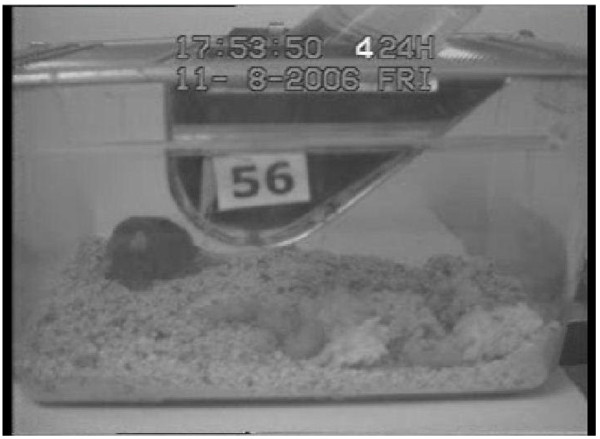
Female lying outside nest in a hunched posture for several hours while the pups were still alive and spread out around nest.

**Figure 5:**
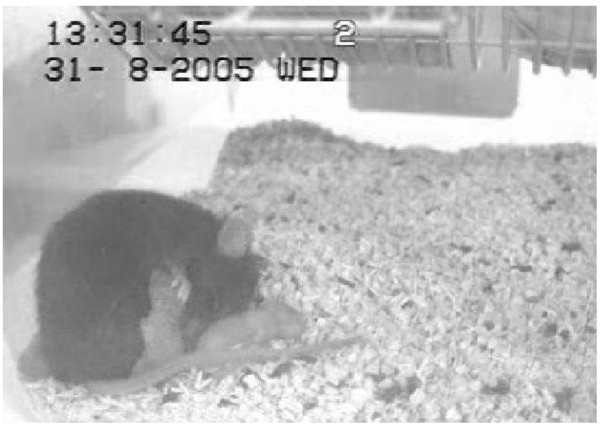
Female manipulating a dead pup in nest.

Several events observed that might be related to pup survival were noted as comments while scoring behaviour. In one female, the first pup was stuck for 1 h in the cervical canal during parturition. This pup was never seen moving and the female did not interact with the pup after it came loose. The female was outside the nest when the parturition started and during the following 30 min. Another female was lying outside the nest for several hours in a hunched posture, while the pups were spread around in the nest and still alive (Figure 4). This female also moved the nest and pups to a new location in the cage about 1.5 day after parturition, but moved it back to the original site 3 h later. Two females were observed performing maternal behaviour with still pups on several occasions (Figure 5). These interactions included licking pup (in mouth and anogenital region), retrieving pup to nest, and manipulating pup with paws. Both these females had pups that were seen moving after birth. During the interactions, one of the females had live pups in the nest while manipulating a dead pup whereas the other female had no moving pups left. Females were also seen eating dead pups in the nest while still having live pups in the nest.

## Discussion

In line with earlier reports, we could observe female mice eating dead offspring. Females did interact with both moving and still pups, but at no instance we observed manipulation of a moving pup being followed by the pup being still. Nor were any wounds on the pups detected. In most cases the pups were active after birth, then displayed successively smaller movements until their activity was very difficult to detect and rarely seen, and the pups were finally lying still not moving anymore. Hence, even when using very detailed observations, we found no evidence that females actively kill their pups. In a study examining infanticide in wild rodents (capybara, *Hydrochaeris hydrochaeris*) bred in captivity for production, the authors could not show that female capybaras killed their offspring [29]; however, the observation method was not described in detail. The farmed mink (*Mustela vison*) is another species with altricial young where high perinatal mortality is common and maternal infanticide has been suggested to be among the main causes [30]. However, in a detailed study on periparturient behaviour, Malmkvist et al. [31] did not find infanticidal mothers. Several experimental studies have shown infanticide in female mice. However, these are experimental studies conducted to observe infanticide from a behavioural ecology perspective where females with differing sexual experience are exposed to related or unrelated pups [for overview [1,27,32]; these studies do not represent normal husbandry conditions and are not applicable when addressing the question about infanticide under the current conditions.

Of the ten females in this study, three had litters in which pups were never seen moving, and in the litters of an additional three females 1–2 pups were never seen moving. This indicates that some pups were most likely dead at birth. The live pups of the remaining seven females did not all stop moving at the same time; thus if any pups were indeed actively killed by the female, the entire litter was not killed at once. Nor did females start eating the pups immediately after they had stopped moving. In most cases the pups were lying still for several hours before the female started eating them.

Apart from being killed by the mother, there are several possible causes for stillbirths which were not further investigated in this study. Mouse pups are also totally dependent on their mother for nutrition and thermoregulation, and might die from starvation or hypothermia if these basic needs are not satisfied [1]. In this study we did not aim to look for underlying causes of litter loss. Instead, we focused on searching for evidence of infanticide, and therefore only mother-pup interactions were observed. The detailed behaviour observations allowed us to identify incidents of problematic parturition which may also be relevant. It is well-known in other mammals (e.g. pigs) that problems during birth are associated with poor neonatal viability [33]. We observed one female with dystocia, another female giving birth to a pup at 8 h after start of parturition, and a third female that was lying still outside the nest in a hunched posture for several hours without physical contact with her live born pups which were spread out in the cage.

Finding the exact time of birth proved to be difficult. It was sometimes impossible to see if a female lying in the nest was still pregnant or if she was nursing, especially in cages with nest material. In some instances it was not possible to see all interactions between female and pups, due to the female turning away from the camera, nest material covering pups or low video quality. For this study, a closer view of the nest site would have allowed us to see more details. On the other hand, mice sometimes move their nest and zooming on a part of the cage might increase the risk that the mice move out of sight. If only parts of the cage are in view, other valuable events might also be missed, such as females lying in a hunched posture outside the nest (might indicate pain), females giving birth outside the nest, pups spread out in the cage, etc. Beside this, mice build elaborate nests, making it difficult to see all details in the nest.

Notably, it was very valuable to be able to access recordings of females that lost their litters. Also with the limitations of the camera switcher recording system, these recordings gave us much information compared to counting pups only at birth and weaning, or even daily inspections. Not only could we observe interactions between mother and pups in detail, we could also detect dystocia, still born pups and females displaying behaviours that seemed to indicate pain. These observations suggest that in the present study there were a number of different causes for pup death, and stress the importance of more careful investigations before assuming that pup death is caused by infanticidal mothers. With normal husbandry routines, pups are generally inspected and counted at first after a couple of days postpartum. However, only by counting pups daily from birth and the first days postpartum, it is possible to assess the number of pups born, and how many that dies before weaning. Inspecting the cage more than once daily further makes it possible to detect stillborn pups, pups with intact amniotic sac and pups with different deformations, before they are being eaten by the mother. Careful inspections several times daily also increase the likelihood of detecting females with problems during or after labour. Our observations indicated that three of the females had indeed difficult parturitions.

It is sometimes recommended to use foster mothers to ensure pup survival in certain transgenic strains and to monitor parturition in especially valuable transgenic strains in order to be able to take rapid action and foster valuable pups in case the parturient female dies or is euthanized. However, monitoring parturitions in general, not only in expensive genetically modified mice, would allow appropriate action to be taken in cases of severe dystocia which if unattended will lead to loss of both female and litter, whereas euthanasia of the female and subsequent fostering of the pups would reduce animal suffering and mortality.

Litter loss in laboratory mice can only be prevented efficiently when the problem is understood and the events leading up to pup death are known. By assuming that pups are killed by the mother, based on the fact that they are found half-eaten or not found at all, the true causes of pup loss are probably overlooked and a welfare problem in laboratory mice is left unresolved.

## Conclusion

In this case study of mother-young behaviour of 10 females losing their litter, we have found no evidence that female laboratory mice kill their pups actively. Rather, our results suggest that other causes than infanticide might play a major role in pup death.

## Competing interests

The authors declare that they have no competing interests.

## Authors’ contributions

All authors contributed to the study design. EW performed all behaviour observations and analysed the data. All authors contributed to the manuscript and have read and approved the final manuscript.
